# Anti-adipogenic Effect of β-Carboline Alkaloids from Garlic (*Allium sativum*)

**DOI:** 10.3390/foods8120673

**Published:** 2019-12-12

**Authors:** Su Cheol Baek, Ki Hong Nam, Sang Ah Yi, Mun Seok Jo, Kwang Ho Lee, Yong Hoon Lee, Jaecheol Lee, Ki Hyun Kim

**Affiliations:** School of Pharmacy, Sungkyunkwan University, Suwon 16419, Korea; schii513@daum.net (S.C.B.); nam6422@hanmail.net (K.H.N.); angelna1023@hanmail.net (S.A.Y.); anstjr920827@gmail.com (M.S.J.); sholaly@naver.com (K.H.L.); yhl2090@naver.com (Y.H.L.)

**Keywords:** *Allium sativum*, β-carboline alkaloids, anti-adipogenic effects, 3T3-L1 preadipocytes, Ac-α-tubulin

## Abstract

Garlic (*Allium sativum* L.) is utilized worldwide for culinary and medicinal use and has diverse health benefits. As part of our ongoing research to identify bioactive components from natural resources, phytochemical analysis of the methanolic extract of garlic led to the isolation and characterization of six compounds: Three eugenol diglycosides (**1**–**3**) and three β-carboline alkaloids (**4**–**6**). In particular, the absolute configurations of β-carboline alkaloids (**5** and **6**) were established by gauge-including atomic orbital nuclear magnetic resonance chemical shift calculations, followed by DP4+ analysis. Here, we evaluated the effects of compounds **1**–**6** on 3T3-L1 preadipocyte adipogenesis and lipid metabolism. 3T3-L1 adipocyte differentiation was evaluated using Oil Red O staining; the expression of adipogenic genes was detected using RT-qPCR. Among compounds **1**–**6**, (1*R,*3*S*)-1-methyl-1,2,3,4-tetrahydro-*β-*carboline-3-carboxylic acid (**6**) inhibited 3T3-L1 preadipocyte adipogenesis and reduced the expression of adipogenic genes (*Fabp4*, *PPARγ*, *C/EBPβ*, *Adipsin*, and *Adipoq*). Moreover, it markedly decreased the actylation of α-tubulin, which is crucial for cytoskeletal remodeling during adipogenesis. Anti-adipogenic effects were observed upon treatment with compound **6,** not only during the entire process, but also on the first two days of adipogenesis. Additionally, treatment with compound **6** regulated the expression of genes involved in adipocyte lipid metabolism, decreasing the lipogenic gene (*SREBP1*) and increasing lipolytic genes (*ATGL* and *HSL*). We provide experimental evidence of the health benefits of using (1*R,*3*S*)-1-methyl-1,2,3,4-tetrahydro-*β-*carboline-3-carboxylic acid obtained from garlic to prevent excessive adipogenesis in obesity.

## 1. Introduction

Garlic (*Allium sativum* L.) is one of the bulbous plants in the family Liliaceae, more recently attributed to the family Amaryllidaceae [1]. Since ancient times and to date, garlic has been a part of people’s lives as a condiment or culinary spice, therapeutic agent against common diseases, cleansing aid, and energy booster for athletes. Interestingly, in ancient Greece and Rome, the consumption of garlic was believed to give courage to soldiers and sailors, respectively. [2]. The most commonly utilized part of garlic is its bulb, which has a characteristic spicy and pungent flavor and is a fundamental component in several cuisines from Southern, Eastern, and Southeastern Asia, Northern Africa, Middle East, and Southern Europe. Since ancient times, garlic has been used in traditional medicine to treat fevers, headaches, intestinal worms, and dysentery [3]. In Asia, garlic is used as a food preservative, a remedy for fever or indigestion, and an antimicrobial agent [4]. Garlic is well-known for its effects against cancer and for increasing immunity; therefore, it is sold as a functional food worldwide [5]. Further, garlic extracts exhibit antifungal, antibacterial, antiviral, antioxidant, immuno-stimulating, and cholesterol-lowering properties [6,7]; the therapeutic functions of garlic in chronic diseases include the prevention of diabetes and platelet aggregation [8,9,10,11]. Several literatures demonstrated the correlation between the consumption of garlic and the decreased risk of cancer development in various organs [12,13,14]. The anticancer potential of garlic has been known to be due to the interaction of bioactive components in garlic with specific molecular targets, which range from cell cycle control to the expression of antioxidants and detoxification enzymes [15,16]. Owing to the health benefits of garlic, its phytochemical constituents have been actively investigated. Garlic is predominantly composed of organosulfur compounds, which give it its pungent smell and spicy taste. A major sulfur-containing compound in garlic is *S*-allyl-L-cysteine sulfoxide (alliin) [17], which forms complexes with the enzyme alliinase, following crushing, cutting, or grinding of garlic bulbs [3]. Alliinase causes the breakdown of alliin to form various volatile sulfur compounds like diallyl sulfide and diallyl disulfide [18,19]. Other reported compounds in the alliums are saponins and polyphenols, which contribute to the bioactivity of allium plant extracts [18,19,20]. In addition, previous phytochemical studies of garlic revealed the presence of eugenol diglycosides and β-carboline alkaloids with biological activities [20,21].

As part of our ongoing research to identify bioactive components from Korean natural resources [22,23,24], we explored the potential bioactive constituents of garlic. The phytochemical analysis of the methanolic (MeOH) extract of garlic led to the isolation of six compounds: Three eugenol diglycosides (**1**–**3**) and three β-carboline alkaloids (**4**–**6**). They were identified via a comparison of their nuclear magnetic resonance (NMR) spectroscopic data with reported values, as well as by LC/MS analysis. Absolute configurations of the β-carboline alkaloids (**5** and **6**) were established using gauge-including atomic orbital (GIAO) NMR chemical shift calculations, followed by DP4+ analysis. Several studies have identified garlic-derived organosulfur compounds that display anti-obesity effects [25,26,27,28]. However, there is limited information about the metabolic effects of other active compounds isolated from garlic compared to that of the organosulfur compounds. In this study, we evaluated the effects of compounds **1**–**6** on the adipogenesis of 3T3-L1 preadipocytes and lipid metabolism, suggesting the potential therapeutic activity of these compounds against obesity and metabolic diseases. Here, we provide details on the extract, isolation, and structural clarity of the compounds, as well as their biological effects on the lipid metabolism of adipocytes.

## 2. Materials and Methods

### 2.1. Extraction of Garlic Sample and Isolation of Compounds

General experimental procedures and information regarding the garlic sample used in this study are included in Supplementary Materials. Minced *A. sativum* (1 kg) was extracted three times with 100% MeOH (18 L) in a day at room temperature and filtered. The resultant filtrate was evaporated using a rotavapor, which gave the MeOH extract (101.7 g). The MeOH extract was successively subjected to solvent-partitioning with *n*-hexane, CH_2_Cl_2_ (MC), ethyl acetate (EA), and *n*-butanol (BuOH), yielding residues weighing 1.4, 0.287, 0.153, and 4.5 g, respectively. A detailed description of the phytochemical analysis and isolation of the six compounds (**1-6**) from *n*-BuOH-soluble fraction are included in Supplementary Materials.

### 2.2. Cell Culture and Differentiation

3T3-L1 preadipocytes were maintained in DMEM containing 10% bovine calf serum (BCS) and 1% P/S. For the differentiation of 3T3-L1 cells into mature adipocytes, the cells were incubated in DMEM with 10% FBS, 1% P/S, 0.5 mM of 3-isobuyl-1-methylxanthine (IBMX), 1 μM of dexamethasone, and 1 μg/mL of insulin. Then, the medium was replaced every 2 days with DMEM with 10% FBS, 1% P/S, and 1 μg/mL of insulin. To assess the effects of compounds **1**–**6** on adipogenesis, we treated 3T3-L1 cells with compounds **1**–**6** during the entire process of adipogenesis. Eight days after starting differentiation (day 8), the cells were harvested and subjected to further experiments, including immunoblotting and RT-qPCR.

### 2.3. Cell Counting

3T3-L1 cells were treated with compounds **1**–**6** for 24 h, then detached from the plate with EDTA. The detached cells were diluted with PBS, and the cell number was counted using LUNA-II™ Automated Cell Counter (Logos Biosystems, Anyang, Korea).

### 2.4. Immunoblotting

Proteins in the adipocytes were extracted using Pro-Prep (Intron Biotechnology, Seongnam, Korea), and 20 μg of each protein was separated by SDS-polyacrylamide gel (12%) electrophoresis. Separated proteins were transferred to polyvinylidene difluoride (PVDF, Millipore, Burlington, MA, USA) membranes via a semi-dry transfer (Bio-Rad, Hercules, CA, USA). The membranes were blocked with non-fat dry milk for 1 h and incubated with the indicated primary antibodies (dilution 1:2000) overnight, followed by incubation with horseradish peroxidase (HRP)-conjugated secondary antibodies for 1 h (Abcam, Cambridge, UK). HRP signals reacting with chemiluminescence reagents (Abclon, Guro, Korea) were detected on AGFA x-ray film CP-Bu NEW and quantified using ImageJ. Anti-acetylated α tubulin (Santa Cruz Biotechnology, SC-23950, Dallas, TX, USA) and anti-tubulin (Santa Cruz Biotechnology, SC-32293, Dallas, TX, USA) were used for the immunoblotting assay.

### 2.5. Reverse Transcription and Quantitative Real-Time PCR (RT-qPCR)

Total RNA from the adipocytes was extracted with Easy-Blue reagent (Intron Biotechnology). Then, cDNA was generated from 1 μg of extracted RNA using the Maxim RT-PreMix Kit (Intron Biotechnology). For quantitative real-time PCR (qPCR), cDNA was mixed with KAPA SYBR^®^ FAST qPCR Master Mix (Kapa Biosystems) and each primer is mentioned below. The qPCR reaction was detected using a CFX96 Touch^TM^ real-time PCR detector (Bio-Rad). Relative mRNA levels for each reaction were normalized to that of *β-Actin*. Relative expression differences were calculated using the ΔΔCT method [29]. The qPCR primer sequences used in this study are listed in Table 1.

### 2.6. Oil Red O Staining

Oil Red O staining was performed to visualize lipid droplets in the adipocytes. Mature adipocytes were fixed with 10% formaldehyde for 1 h and washed with 60% isopropanol. Then, the cells were incubated with the Oil Red O working solution for 1 h, followed by washing twice with distilled water. To prepare the Oil Red O stock solution, 300 mg of Oil Red O powder was dissolved in 100 mL of 99% isopropanol. The Oil Red O working solution, prepared just before use, contained three parts of Oil Red O stock solution and two parts of distilled water.

### 2.7. Statistical Analysis

Statistical significance was evaluated using Student’s two-tailed t-test with Excel and assessed based on the *p*-value. Data represent the means ± SEM for n = 3. * *p* < 0.05, ** *p* < 0.01, and *** *p* < 0.001.

## 3. Results and Discussion

### 3.1. Identification of Compounds

**Figure 1:**
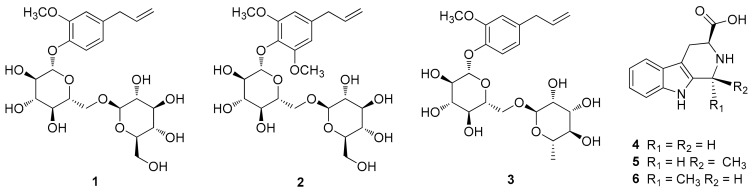
Chemical structures of compounds **1**–**6**.

**Figure 2:**
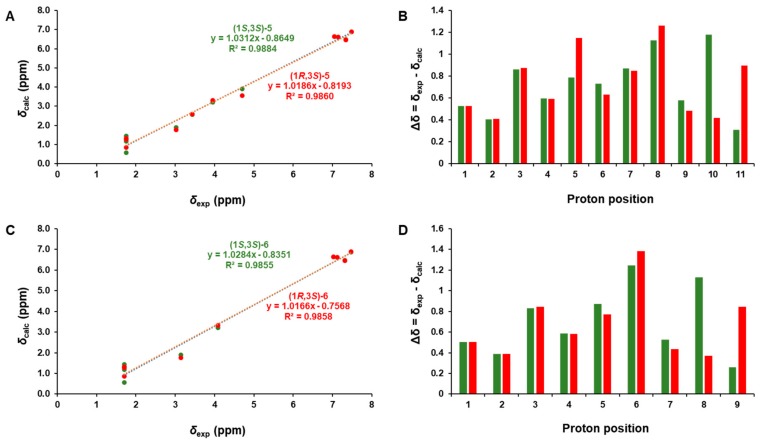
(**A**) Regression analysis of experimentally versus computationally calculated ^1^H NMR chemical shifts of (1*S,*3*S*)-**5** and (1*R,*3*S*)-**5** with linear fitting shown as a line. (**B**) Relative chemical shift errors between calculated and experimental ^1^H NMR data for (1*S,*3*S*)-**5** and (1*R,*3*S*)-**5**. (**C**) The regression analysis of experimentally versus computationally calculated ^1^H NMR chemical shifts of (1*S,*3*S*)-**6** and (1*R,*3*S*)-**6** with linear fitting is shown as a line. (**D**) Relative chemical shift errors between computationally calculated and experimental ^1^H NMR data for (1*S,*3*S*)-**6** and (1*R,*3*S*)-**6**.

The phytochemical analysis of the *n*-BuOH-soluble fraction from the methanol extract of garlic was performed using column chromatography and HPLC purification along with LC/MS-based analysis. The analysis led to the isolation of six compounds (**1**–**6**), including three eugenol diglycosides (**1**–**3**) and three β-carboline alkaloids (**4**–**6**) (Figure 1). The compounds were identified as eugenyl-*O-β-*D-glucopyranosyl-(1→6)-*β-*D-glucopyranoside (**1**) [30], 4-*O-β-*D-glucopyranosyl(1→6)-*β*-D-glucopyranosyl 5-methoxy eugenol (**2**) [30], eugenyl *O-α-*D-rhamnopyranosyl-(1→6)-*β-*D-glucopyranoside (**3**) [31], (3*S*)-1,2,3,4-tetrahydro-*β*-carboline-3-carboxylic acid (**4**) [32], (1*S,*3*S*)-1-methyl-1,2,3,4-tetrahydro-*β*-carboline-3-carboxylic acid (**5**) [32], and (1*R,*3*S*)-1-methyl-1,2,3,4-tetrahydro-*β-*carboline-3-carboxylic acid (**6**) [32], by comparing their NMR spectroscopic data with reported values as well as by LC/MS analysis. In particular, the absolute configurations of β-carboline alkaloids (**5** and **6**) were established by GIAO NMR chemical shift calculations, followed by DP4+ analysis [33]. Given that the β-carboline alkaloids (**5** and **6**) were likely biosynthetically produced from L-tryptophan, a naturally occurring product, the computationally calculated ^1^H NMR chemical shifts of two possible diastereomers (1*S,*3*S*)-**5/6** and (1*R,*3*S*)-**5/6** were compared with the experimental values of **5** and **6** using the results of the DP4+ analysis. The comparison indicated a structural equality of **5** to (1*S,*3*S*)-**5** with 98.54% probability and **6** to (1*R,*3*S*)-**6** with 99.46% probability (Figures S1 and S2). Further, according to the formula Δδ = δ_calcd_ − δ_exptl_, differences (Δδ) were calculated (Tables S1 and S2). The correlation coefficient (*R*^2^) from linear regression analysis, largest absolute deviation (LAD), and the mean absolute deviation (MAD) for the ^1^H NMR data of (1*S,*3*S*)-**5** were 0.9884 (Figure 2A), 1.18, and 0.22 (Figure 2B), respectively, whereas *R*^2^, LAD, and MAD values for the data of (1*R,*3*S*)-**5** were 0.9860, 1.26, and 0.25, respectively. These obtained data supported the result from DP4+ probability analysis to be (1*S,*3*S*) in **5**. In the case of **6**, *R*^2^, LAD, and MAD for ^1^H NMR data of (1*S,*3*S*)-**6** were 0.9855 (Figure 2C), 1.38, and 0.25 (Figure 2D), respectively, whereas *R*^2^, LAD, and MAD values for the data of (1*R,*3*S*)-**6** were 0.9858, 1.25, and 0.28, respectively. Accordingly, the absolute configuration of **6** was assigned to be (1*R,*3*S*), which was in agreement with the DP4+ probability analysis of **6**.

### 3.2. Effects of Compounds 1–6 on Adipogenesis

**Figure 3:**
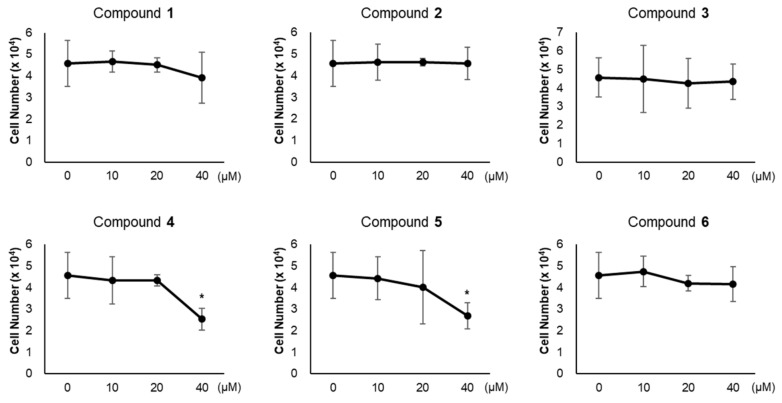
Cytotoxicity of compounds **1**–**6**. Cell viability in 3T3-L1 treated with compounds **1**–**6** (10, 20, 40 μM) for 24 h were determined. Data represent the means ± SD for n = 3. * *p* < 0.05.

**Figure 4:**
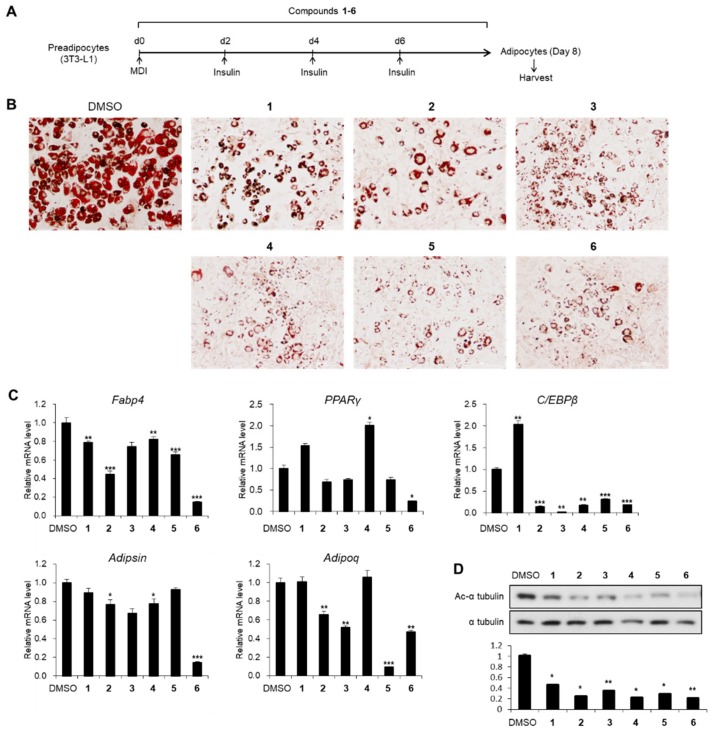
The inhibitory effects of compounds **1**–**6** on adipogenesis. (**A**) Schematic representation of 3T3-L1 differentiation into adipocytes. Cells were treated with compounds **1**–**6** during the entire process of differentiation. (**B**) The Oil Red O staining of 3T3-L1 adipocytes incubated with compounds **1**–**6** (20 μM) during adipogenesis. (**C**) The mRNA levels of *Fabp4*, *PPARγ*, *C/EBPβ*, *Adipsin*, and *Adipoq* genes in 3T3-L1 adipocytes incubated with compounds **1**–**6** (20 μM) during adipogenesis. Data represent the means ± SEM for n = 3. * *p* < 0.05, ** *p* < 0.01, and *** *p* < 0.001. (**D**) Immunoblot analysis of 3T3-L1 adipocytes incubated with compounds **1**–**6** (20 μM) during adipogenesis.

To evaluate the influence of compounds **1**–**6** on adipogenesis, we first assessed the cytotoxicity of compounds **1**–**6** in 3T3-L1 preadipocytes. All compounds exhibited no cytotoxic effects up to 20 μM, but 40 μM of compounds **4** and **5** reduced the viability of 3T3-L1 cells (Figure 3). Thus, we treated 3T3-L1 cells with 20 μM of compounds **1**–**6** during adipogenesis (Figure 4A). The staining of lipid droplets with Oil Red O solution showed that compounds **1**–**6** inhibited lipid droplet accumulation in the adipocytes (Figure 4B). The transcription levels of the adipocyte marker genes (*Fabp4*, *PPARγ*, *C/EBPβ*, *Adipsin*, and *Adipoq*) were reduced upon treatment with compounds **1**–**6** (Figure 4C). During the maturation of adipocytes, the accumulation and fusion of lipid droplets accompany microtubule-dependent cytoskeletal reorganization facilitated by the acetylation of α-tubulin [34]. Thus, we examined whether compounds **1**–**6** influenced the level of the acetylated α-tubulin. As expected, the acetylation of α-tubulin was ablated upon exposure to compounds **1**–**6** during adipogenesis (Figure 4D), implying the failure of cytoskeletal remodeling required for adipogenesis. Among compounds **1**–**6**, compound **6** exhibited the highest anti-adipogenic effect (Figure 4); this enabled us to focus on the activity of compound **6**.

### 3.3. Effects of Compound 6 on the Early Stages of Adipogenesis

**Figure 5:**
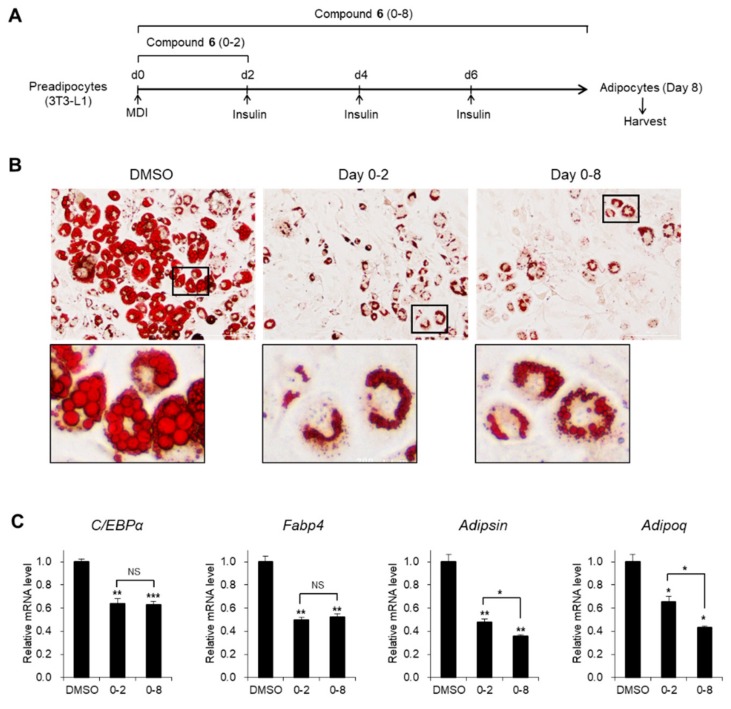
Inhibitory effects of compound **6** on the early stages of adipogenesis. (**A**) Schematic representation of 3T3-L1 differentiation into adipocytes. Cells were treated with compound **6** during the early days (day 0–2) or the entire process of differentiation. (**B**) Oil Red O staining of 3T3-L1 adipocytes incubated with compound **6** (20 μM) during adipogenesis. (**C**) The mRNA levels of *C/EBPα*, *Fabp4*, *Adipsin*, and *Adipoq* genes in 3T3-L1 adipocytes incubated with compound **6** (20 μM) during adipogenesis. Data represent the means ± SEM for n = 3. * *p* < 0.05, ** *p* < 0.01, and *** *p* < 0.001. NS: not significant.

As cytoskeletal changes are early events during adipogenic differentiation [35], we hypothesized that the anti-adipogenic activity of (1*R,*3*S*)-1-methyl-1,2,3,4-tetrahydro-*β-*carboline-3-carboxylic acid (**6**) would be critical in the early stages of adipogenesis (Figure 5A). Treatment with compound **6** during the first 2 days interfered with the generation of adipocytes containing large lipid drops to a level comparable to that of the entire process of treatment with compound **6** (Figure 5B). Magnified images showed that lipid accumulation and fusion were considerably impaired with a big nucleus in the center of the cells upon exposure to compound **6**, whereas most of the cytosolic parts of the control cells were occupied by lipids with a peripheral nucleus (Figure 5B). Inhibitory effects on adipogenic gene expression were consistently observed upon treatment with compound **6** during the early stages as well as the entire process of adipogenesis (Figure 5C). These data indicate that the early stages of adipogenesis were impaired by compound **6**.

### 3.4. Effects of Compound 6 on Lipid Metabolism

**Figure 6:**
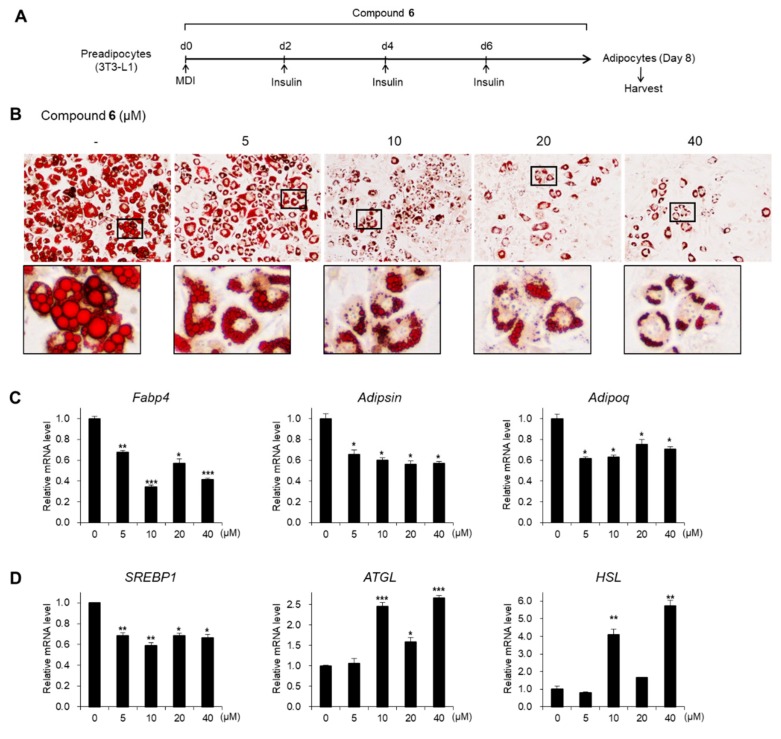
Effects of compound **6** on adipogenesis and lipid metabolism. (**A**) Schematic representation of 3T3-L1 differentiation into adipocytes. Cells were treated with compound **6** during the entire process of differentiation. (**B**) The Oil Red O staining of 3T3-L1 adipocytes incubated with compound **6** during adipogenesis. (**C**) The mRNA levels of *Fabp4*, *Adipsin*, and *Adipoq* genes in 3T3-L1 adipocytes incubated with compound **6** during adipogenesis. (**D**) The mRNA levels of *SREBP1*, *ATGL*, and *HSL* genes in 3T3-L1 adipocytes incubated with compound **6** during adipogenesis. Data represent the means ± SEM for n = 3. * *p* < 0.05, ** *p* < 0.01, and *** *p* < 0.001.

Next, we assessed the effects of compound **6** under diverse concentrations (5, 10, 20, and 40 μM) on adipogenesis (Figure 6A). As detected by the Oil Red O staining, adipogenesis and lipid accumulation were markedly inhibited by treatment with compound **6,** even at a low concentration (5 μM; Figure 6B). Moreover, the expression levels of adipogenic genes were significantly reduced upon incubation with a low concentration of compound **6** (Figure 6C), suggesting that compound **6** is a potent modulator of adipogenesis. As the magnified images of stained adipocytes showed lipid dispersion of compound **6** compared to the control (Figure 5B and Figure 6B), we evaluated the capacity of compound **6** to regulate lipid metabolism. The mRNA expression of lipogenic gene *SREBP1* was significantly reduced upon exposure to compound **6** during the maturation of adipocytes, whereas the transcription of lipolytic genes, *ATGL* and *HSL*, was elevated (Figure 6D). These data demonstrate that the interruption of lipid drop enlargement by compound **6** was mediated by the regulation of the gene involved in the lipid metabolism.

**Figure 7:**
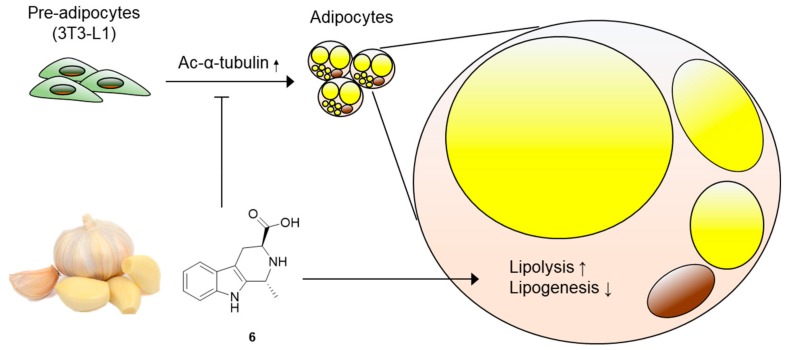
Molecular model underlying the mechanism of action of compound **6**. Compound **6** inhibits adipogenesis from 3T3-L1 preadipocytes, suppresses acetylation of α-tubulin, and regulates lipid metabolism, enhancing lipolysis and reducing lipogenesis.

Obesity, a major cause of diverse metabolic disorders including type-2 diabetes, is characterized by the excessive expansion of white adipose tissue [36]. This process is accompanied by the differentiation of precursor cells into adipocytes (adipogenesis) and lipid drop accumulation in adipocytes (lipogenesis) [37]. Therefore, a pharmacological approach to block adipogenesis and lipogenesis has been considered effective to ameliorate metabolic disorders. In this study, we screened the effects of compounds **1**–**6** obtained from garlic on adipocyte differentiation and identified that (1*R,*3*S*)-1-methyl-1,2,3,4-tetrahydro-*β-*carboline-3-carboxylic acid (compound **6**) can disrupt lipid accumulation in adipocytes by promoting lipolysis and disturbing lipogenesis (Figure 7). While there is abundant evidence demonstrating the neuroprotective and anticancer effects of bioactive *β-*carbolines from diverse natural products [38,39,40,41], the metabolic effects of *β-*carbolines are not well defined. To the best of our knowledge, we report, for the first time, the effects of garlic-derived *β-*carboline alkaloids on adipogenesis and lipid metabolism.

In the early stages of adipogenic differentiation from preadipocytes, the acetylation of α-tubulin mediates cytoskeletal remodeling and elongation of primary cilia [34,42]. A defect in the acetylation of α-tubulin results in the disappearance of primary cilia, which impairs the generation of mature adipocytes [42,43]. Treatment with compound **6** effectively disturbed the acetylation of α-tubulin during adipogenesis, which is apparently responsible for the anti-adipogenic activity of compound **6**. These data offer better understanding of the manner in which compound **6** prevents adipogenesis from 3T3-L1 cells.

## 4. Conclusions

We provide experimental evidence of the potential role of (1*R,*3*S*)-1-methyl-1,2,3,4-tetrahydro-*β-*carboline-3-carboxylic acid (**6**) in adipogenesis from preadipocytes. The active compound was isolated from garlic (*A. sativum*), one of the most popular seasoning agents or condiments used worldwide; it is a β-carboline alkaloid, which is not a common constituent of garlic. Compound **6** repressed adipocyte differentiation from 3T3-L1 preadipocytes by preventing cytoskeletal remodeling, which is essential for adipogenesis. Moreover, compound **6** inhibited lipid accumulation by regulating lipolytic and lipogenic gene expression. Our findings provide a potential therapeutic strategy that uses a novel active compound from garlic to prevent excessive adipogenesis in obesity.

## Figures and Tables

**Table 1:** Sequences of primers for mRNA used in RT-qPCR.

Gene	Forward	Reverse
*β-Actin*	5′-ACGGCCAGGTCATCACTATTG-3′	5′-TGGATGCCACAGGATTCCA-3′
*Adipsin*	5‘-CATGCTCGGCCCTACATG-3′	5‘-CACAGAGTCGTCATCCGTCAC-3′
*Adipoq*	5′-TGTTCCTCTTAATCCTGCCCA-3′	5′-CCAACCTGCACAAGTTCCCTT-3′
*ATGL*	5′-TTCACCATCCGCTTGTTGGAG-3′	5′-AGATGGTCACCCAATTTCCTC-3′
*C/EBPα*	5′-CTCCCAGAGGACCAATGAAA-3′	5′-AAGTCTTAGCCGGAGGAAGC-3′
*C/EBPβ*	5′-GGACAAGCTGAGCGACGAGTA-3′	5′-CAGCTGCTCCACCTTCTTCTG-3′
*Fabp4*	5‘-AAGGTGAAGAGCATCATAACCCT-3′	5‘-TCACGCCTTTCATAACACATTCC-3′
*HSL*	5′-CACAAAGGCTGCTTCTACGG-3′	5′-GGAGAGAGTCTGCAGGAACG-3′
*PPARγ*	5‘-GCATGGTGCCTTCGCTGA-3′	5‘-TGGCATCTCTGTGTCAACCATG-3′
*SREBP1*	5′-AACGTCACTTCCAGCTAGAC-3′	5′-CCACTAAGGTGCCTACAGAGC-3′

## Supplementary Materials

The following are available online at https://www.mdpi.com/2304-8158/8/12/673/s1, Figure S1: DP4+ analysis of compound **5** with isomers (1*S*,3*S*)-**5** (Isomer 1) and (1*R*,3*S*)-**5** (Isomer 2), Figure S2: DP4+ analysis of compound **6** with isomers (1*S*,3*S*)-**6** (Isomer 1) and (1*R*,3*S*)-**6** (Isomer 2), Table S1: The computed ^1^H NMR data for (1*S*,3*S*)-**5** and (1*R*,3*S*)-**5**, Table S2: The computed ^1^H NMR data for (1*S*,3*S*)-**6** and (1*R*,3*S*)-**6**. General experimental procedures, plant material, extraction and isolation, computational NMR chemical shift calculation procedures for DP4+ analysis, DP4+ analysis results of compounds **5** and **6**, and the computed ^1^H NMR data for (1*S*,3*S*)-**5**/**6** and (1*R*,3*S*)-**5**/**6** are available free of charge on the internet.
