# Supplementary material for: Anti-adipogenic Effect of β-Carboline Alkaloids from Garlic (Allium sativum)
Source: Foods. 2019 Dec 12;8(12):673. doi: 10.3390/foods8120673 (PMC6963209; doi:10.3390/foods8120673)
Supplement: Supplementary file 1 [file foods-08-00673-s001.pdf]

Supplementary Materials

Anti-adipogenic effect of β-carboline Alkaloids from garlic (*Allium sativum*)

Su Cheol Baek †, Ki Hong Nam †, Sang Ah Yi †, Mun Seok Jo, Kwang Ho Lee, Yong Hoon Lee, Jaecheol Lee *, and Ki Hyun Kim *

School of Pharmacy, Sungkyunkwan University, Suwon 16419, Republic of Korea

* Correspondence: jaecheol@skku.edu (J.L.); khkim83@skku.edu (K.H.K.);Tel.: +82-31-290-7726 (J.L.); +82-31-290-7700 (K.H.K.)

† These authors contributed equally to this work.

Figure S1. DP4+ analysis of compound **5** with isomers (1*S*,3*S*)-**5** (Isomer 1) and (1*R*,3*S*)-**5** (Isomer 2).

Functional		Solvent?		Basis Set		Type of Data	
B3LYP		PCM		6-31G(d)		Unscaled Shifts	
		DP4+	98.54%	1.46%	-	-	-
Nuclei	sp2?	Experimental	Isomer 1	Isomer 2	Isomer 3	Isomer 4	Isomer 5
H	x	7.14	6.6	6.6			
H	x	7.05	6.6	6.6			
H	x	7.34	6.5	6.5			
H	x	7.48	6.9	6.9			
H		4.7	3.9	3.6			
H		3.95	3.2	3.3			
H		3.44	2.57	2.6			
H		3.02	1.89	1.8			
H		1.75	1.172685634	1.27			
H		1.75	0.568769511	1.33			
H		1.75	1.441724664	0.85			

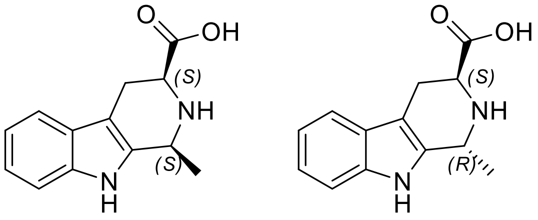

(1*S*,3*S*)-**5** (1*R*,3*S*)-**5**

Figure S2. DP4+ analysis of compound **6** with isomers (1*S*,3*S*)-**6** (Isomer 1) and (1*R*,3*S*)-**6** (Isomer 2).

Functional		Solvent?		Basis Set		Type of Data	
B3LYP		PCM		6-31G(d)		Unscaled Shifts	
		DP4+	0.54%	99.46%	-	-	-
Nuclei	sp2?	Experimental	Isomer 1	Isomer 2	Isomer 3	Isomer 4	Isomer 5
h	x	7.12	6.6	6.6			
h	x	7.03	6.6	6.6			
h	x	7.31	6.5	6.5			
h	x	7.47	6.9	6.9			
h		4.09	3.2	3.3			
h		3.14	1.9	1.8			
h		1.7	1.2	1.3			
h		1.7	0.6	1.3			
h		1.7	1.4	0.9			

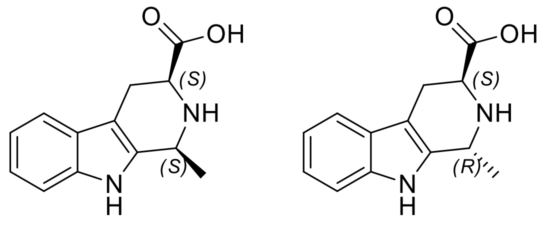

(1*S*,3*S*)-**6** (1*R*,3*S*)-**6**

Table S1. The computed ^{1}H NMR data for (1*S*,3*S*)-**5** and (1*R*,3*S*)-**5**.

No.	5	δ_{exp}	(1*S*,3*S*)-**5**		(1*R*,3*S*)-**5**	
			δ_{cal} (ppm)	$\Delta\delta$	δ_{cal} (ppm)	$\Delta\delta$
**1**	6	7.14	6.61	0.53	6.61	0.53
**2**	7	7.05	6.64	0.41	6.64	0.41
**3**	8	7.34	6.48	0.86	6.46	0.88
**4**	5	7.48	6.88	0.60	6.89	0.59
**5**	1	4.7	3.91	0.79	3.55	1.15
**6**	3	3.95	3.22	0.73	3.32	0.63
**7**	4'	3.44	2.57	0.87	2.59	0.85
**8**	4''	3.02	1.89	1.13	1.76	1.26
**9**	10	1.75	1.17	0.58	1.27	0.48
**10**	10	1.75	0.57	1.18	1.33	0.42
**11**	10	1.75	1.44	0.31	0.85	0.90
**MAD**[b]			0.22		0.25	
**LAD**[a]			1.18		1.26	

[a]**LAD = largest absolute deviation.**

[b]**MAD = mean absolute deviation, computed as (1/*n*)** $\sum_i^n \left|\delta_{calcd} - \delta_{exptl}\right|$

Table S2. The computed ^{1}H NMR data for (1*S*,3*S*)-**6** and (1*R*,3*S*)-**6**.

**No.**	**6**	δ_{exp}	(1*R*,3*S*)-**6**		(1*S*,3*S*)-**6**	
			δ_{cal} (ppm)	$\Delta\delta$	δ_{cal} (ppm)	$\Delta\delta$
**1**	6	7.12	6.61	0.51	6.61	0.51
**2**	7	7.03	6.64	0.39	6.64	0.39
**3**	8	7.31	6.48	0.83	6.46	0.85
**4**	5	7.47	6.88	0.59	6.89	0.58
**5**	3	4.09	3.22	0.87	3.32	0.77
**6**	4''	3.14	1.89	1.25	1.76	1.38
**7**	10	1.7	1.17	0.53	1.27	0.43
**8**	10	1.7	0.57	1.13	1.33	0.37
**9**	10	1.7	1.44	0.26	0.85	0.85
**MAD**[b]			0.28		0.25	
**LAD**[a]			1.25		1.38	

[a]**LAD = largest absolute deviation.**

[b]**MAD = mean absolute deviation, computed as (1/*n*)** $\sum_i^n |\delta_{calcd} - \delta_{exptl}|$

General experimental procedures

Optical rotations were calculated using a Jasco P-1020 polarimeter (Jasco, Easton, MD, USA); ultraviolet (UV) spectra were acquired on an Agilent 8453 UV-visible (UV-Vis) spectrophotometer (Agilent Technologies, Santa Clara, CA, USA). The NMR spectra were recorded on a Bruker AVANCE III 800 NMR spectrometer with a 5-mm TCI CyroProbe operating at 800 MHz (^{1}H), with chemical shifts given in ppm (δ) (Bruker). Preparative high-performance liquid chromatography (HPLC) was performed using a Waters 1525 Binary HPLC pump with a Waters 996 photodiode array detector (Waters Corporation Milford, MA, USA) and an Agilent Eclipse C_{18} column (250 × 21.2 mm, 5 μm; flow rate: 5 mL/min; Agilent Technologies). Semi-preparative HPLC was performed using a Shimadzu Prominence HPLC System with SPD-20A/20AV Series Prominence HPLC UV-Vis Detectors (Shimadzu, Tokyo, Japan). The LC/MS analysis was performed on an Agilent 1200 Series HPLC system equipped with a diode array detector and a 6130 Series electrospray ionization mass spectrometer using an analytical Kinetex® 5-μm C_{18} 100 Å column (5 μm, 2.1 × 100 mm, Phenomenex, Torrance, CA, USA). Column chromatography was performed with Silica gel 60 (Merck, Darmstadt, Germany; 230–400 mesh) and reverse-phase (RP)-C_{18} silica gel (Merck, 230–400 mesh). The packing material for the molecular sieve column chromatography was Sephadex LH-20 (Pharmacia, Uppsala, Sweden). Precoated silica gel F_{254} plates and RP-18 F_{254s} plates (Merck) were used for thin-layer chromatography (TLC). Spots were detected on TLC under UV light or by heating after spraying with anisaldehyde-sulfuric acid.

Plant material

Allium sativum L. was collected from Uiseong, Gyeongsangbuk-do, Korea, in March 2016. The material was identified by one of

the authors (K. H. Kim). A voucher specimen (MN-16-03) was deposited in the herbarium of the School of Pharmacy, Sungkyunkwan University

Extraction and isolation

Minced *A. sativum* (1 kg) was extracted with 100% MeOH (18 L × 1 day × three times) at room temperature and filtered. The resultant solution was evaporated under reduced pressure using a rotavapor to obtain the MeOH extract (101.7 g), which was suspended in distilled water (1.4 L) and successively solvent-partitioned with *n*-hexane, CH_2Cl_2 (MC), ethyl acetate (EA), and *n*-butanol (BuOH), yielding residues weighing 1.4 g, 0.287 g, 0.153 g, and 4.5 g, respectively. The *n*-BuOH-soluble fraction (4.5 g) was subjected to silica gel open column chromatography using a gradient solvent system of CH_2Cl_2/methanol (MeOH) (10:1), CH_2Cl_2/MeOH/H_2O (9:3:0.5), and 100% MeOH to obtain seven fractions (B1–B7). Fraction B5 (250.7 mg) was separated by preparative reversed-phase HPLC with a gradient solvent system of MeOH/H_2O (9:1 to 1:0) to obtain five subfractions (B5a–B5e). Subfraction B5e (60 mg) was purified using semi-preparative HPLC with a solvent system of MeOH/H_2O (41:59) to yield compounds **1** (2.1 mg), **2** (1.6 mg), and **3** (1.1 mg). Fraction B6 (665.9 mg) was separated using Sephadex LH-20 open column chromatography with a solvent system of 100% MeOH to obtain five subfractions (B6a–B6e). Subfraction B6e (180.8 mg) was separated by preparative reversed-phase HPLC with a solvent system of MeOH/H_2O (3:7 to 1:0) to afford four subfractions (B6e-1–B6e-4). Subfraction B6e-2 (69.2 mg) was purified using semi-preparative HPLC with a solvent system of MeOH/H_2O (1:3) to yield compounds **4** (2.3 mg), **5** (2.3 mg), and **6** (1.2 mg).

Computational NMR chemical shift calculations for DP4+ analysis

Conformational searches were performed using Tmolex 4.3.1 with the DFT settings (B3-LYP functional/M3 grid size), geometry optimization settings (energy 10^{-6} hartree, gradient norm $|dE/dxyz| = 10^{-3}$ hartree/bohr), and the basis set def-SV(P) for all atoms. The NMR shielding constants were calculated on optimized ground state geometries at the DFT B3LYP/def-SV(P) level of theory. The NMR chemical shifts of the isomers were obtained by Boltzmann averaging the ^{1}H NMR and ^{13}C NMR chemical shifts of the stable conformers at 298.15 K. The chemical shift values were calculated using

$$\delta_{calc}^{x} = \frac{\sigma^{o} - \sigma^{x}}{1 - \sigma^{o}/10^{6}};$$

where, δ_{calc}^{x} is the calculated NMR chemical shift for nucleus x and σ^{o} is the shielding tensor for the proton and carbon nuclei in tetramethylsilane calculated at the DFT B3LYP/def-SV(P) basis set [1].

The calculated NMR properties of the optimized structures were averaged based on their respective Boltzmann populations, and the DP4+ probability analysis was facilitated by the Excel sheet (DP4+) provided by Grimblat et al. [2].

Reference)

[1] Smith, S.G.; Goodman, J. M.; Assigning stereochemistry to single diastereoisomers by GIAO NMR calculation: the DP4 probability. *J. Am. Chem. Soc*. **2010**, *132*, 12946–12959.

[2] Grimblat, N.; Zanardi, M. M.; Sarotti, A. M.; Beyond DP4: an improved probability for the stereochemical assignment of isomeric compounds using quantum chemical calculations of NMR shifts. *J. Org. Chem*. **2015**, *80*, 12526-12534.
